# Experimental studies on the effect of (Lambda-Cyhalothrin) insecticide on lungs and the ameliorating effect of plant extracts (Ginseng (Panax Ginseng) and garlic (Allium sativum L.) on asthma development in albino rats

**DOI:** 10.1186/1756-0500-7-243

**Published:** 2014-04-16

**Authors:** Mouchira M Mohi El-Din, Amna M Mostafa, Aml Abd-Elkader

**Affiliations:** 1 Pathology and Clinical Pathology Department, Faculty of Veterinary Medicine, South Valley University, 83523 Qena, Egypt; 2 Zoo Department, Science Faculty, South Valley University, 83523 Qena, Egypt

**Keywords:** Albino rats, Airway inflammation, Asthma, LTC, Garlic, Ginseng plants, Pathology

## Abstract

**Background:**

Lambda-cyhalothrin (LTC) is a synthetic pyrethroid insecticide for agricultural and public health applications. This study was to determine the pathological alterations of LTC in lungs, which has not previously been studied, and the ameliorating effects of plant extracts (ginseng and garlic) on the development of asthma in albino rats.

**Methods:**

Four groups (gps) of albino rats, (n = 20, average body weight = 200 gm with an age of 4 months), were formed. Gp 1 was kept as control. Gp 2 was injected intraperitoneally (i.p.) with LTC at a dose of 1/6 LD50 that is 9.34 mg/kg body weight (w.t.) daily for 21 days (d). Gp 3 & 4 were injected (i.p.) with ginseng at the dose of 200 mg/kg b.wt and garlic (Allium sativum L.) at the dose of 100 mg/kg b.wt., respectively, one hour before being given LTC at a dose of 1/6 LD50 (9.34 mg/kg b.wt.) daily. Each groups were divided into two sacrificed, at 15 and 21 d p.i. Blood and lung samples were collected for hematological and histopathological examinations.

**Results:**

Hematological findings showed that the animals in gps 2 and 3, which were treated for 21 days, showed a significant difference in RBC counts (P > .001), Hb (P > .007), PCV% (P > .004), (P > .008) in comparison with the control group. Signs of cough and nasal discharge were seen in gp 2, which became mild in gp 4. Grossly, the lungs showed congestion and consolidation in gp 2. Histopathologically, macroabscesses and interstitial alveolitis were seen in gp 2, which led to obstruction in the lumen of the bronchioles at 21 d p.i. Meanwhile, thickening in the interalveolar septa with mononuclear cells was seen in gps. 3 and 4 at 21d p.i.

**Conclusions:**

The study shows 3 gps of rats injected with LHC alone or combined with garlic and ginseng extract, each group were divided into two sacrificed (15 and 21 d p.i.). Lambda cyhalothrin causes bronchial obstruction in the lungs of the rats (15 and 21 d p.i), which decreased into mild to moderate interstitial inflammation in the rats given garlic and ginseng, respectively.

## Background

Lambda-cyhalothrin (LTC) is a synthetic pyrethroid insecticide for agricultural and public health applications. Pyrethroid insecticides are manufactured chemicals that are very similar in structure to pyrethrins, but more toxic to insects as well as to mammals, and last longer in the environment than pyrethrins. These pesticides have injurious effects, including damage to the lungs and dysfunctioning of the immune and endocrine systems [1].

Asthma is a chronic disease that affects airways carrying air in and out of the lungs. The internal wall of the asthmatic airways become inflamed, swollen and very sensitive and reactive, resulting in narrowing of the airways and a lessening of airflow into lung tissues. Asthma is characterized by eosinophilic and non-eosinophilic phenotypes and its characteristics are based on inflammatory cell patterns in airway secretions. Neutrophils are important in innate immunity, and exist excessively in the airways in non-eosinophilic asthma [2]. There have been reports asserting a potential association between exposure to pyrethrins/pyrethroid and allergic/asthmatic effects [3]. Not all pesticides cause asthma, but some synthetic pyrethroids (Permethrin, Cypermethrin, Cyfluthrin, Sumithrin, Resmethrin) cause hypersensitization, asthma, emphysema, or hyperactive airway disease due to overexposure [4].

Panax ginseng is the most famous among Asian medicinal plants. It increases body resistance to many harmful factors and protects tissues from damage [5]. Ginsenosides (from ginseng leaf-stem) have been shown to lower serum lipid, regulate lipid metabolism and promote anti-oxidation, as well as enhance immune activity [6].

Garlic (Allium Sativum L.) was an important medical plant for the ancient Egyptians, as listed in the medical text Codex Ebers (ca. 1550 BC), especially for the working class involved in heavy labor, since it was an effective remedy for many ailments. Garlic confers many therapeutic benefits. Garlic’s strong odor is largely due to sulphur-containing compounds (e.g. S-allylcysteine sulphoxide), which are believed to account for most of its medicinal properties [7].

The aim of this study was to investigate pathological alterations caused by LTC in the lungs and the beneficial effects of two plant extracts [ginseng and garlic (Allium Sativum L.)] on the development of asthma in albino rats.

## Methods

### Chemicals

Lambda-cyhalothrin (lambada super fog 5%) was purchased from ADWIA industries company, Egypt. The animals were given 1/6 of (56 mg/kg LD_50_ for female rats) According to [8].

Dry aged native garlic (2 kg) was prepared for aqueous aged garlic extract. The fresh garlic cloves were peeled on crushed ice and 50 g of garlic was homogenized in 75 ml of cold, sterile 0.9% saline in the presence of some crushed ice. The filtered homogenized mixture was then centrifuged at 2000 round/minute for 5 min. The clear supernatant was made up to 100 ml with normal saline. The concentration of the garlic preparation was considered to be 500 mg/ml. The prepared garlic extract was stored at -20˚C until use. The garlic extract was prepared according to the method reported by [9].

Panax Ginseng (G115) was purchased from EIPICO Pharmaceutical Company, Egypt. Each capsule containing 100 mg of ginseng extract and dissolved in 20 ml of warm saline solution 1 ml of this solution containing (40) mg of ginseng according to [10].

### Experimental animals

The present study was carried with eighty adult female albino rats weighing 200 ± 20 gm at age 4 months. The animals were obtained from the Abu Rawash Breeding House, Giza, Egypt. The animals were housed 3 rats in each cage and examined 2 weeks prior to the experiment. They were kept under normal environmental conditions of temperature and humidity. Commercial standard diet and water were continuously and regularly supplied ad libitum throughout the experimental period. Experiments were carried out in compliance with the guidelines of the Ethical Principles in Animal Research adopted by Ethics of animal use in research committee (EAURC), Vet. Med. College, Cairo University, Egypt (protocol no. 07/ 2013).

### Experimental design

80 adult female albino rats were divided equally into four groups (n = 20). Group (1) the rats were injected intraperitoneally (i.p.) with normal saline and used as a control. Group 2 was injected intraperitoneally (i.p.) with LTC at a dose of 1/6 LD50 that is 9.34 mg/kg body weight (w.t.), dissolved in normal saline daily for 21 days (d). Gp 3 & 4 were injected (i.p.) with ginseng at the dose of 200 mg/kg b.wt and garlic (Allium sativum L.) at the dose of 100 mg/kg b.wt., respectively, one hour before being given LTC at a dose of 1/6 LD50 (9.34 mg/kg b.wt.) daily.

The animals in all groups were examined daily during the course of the experiment, and clinical signs and mortalities were recorded. Two animals died from gp.2 (LTC), one at the 12th d p.i. and the other at the 17th d p.i. The half animals in all groups were sacrificed, at 15 days post-injection and the remaining surviving animals were sacrificed 24 hours after the last treatment dose, at 21 days post-injection.

### Hematological analysis

Blood samples, the animals were starved overnight for 12 h before the blood was collected. Rats were anaesthetized with ether and blood collected from the orbital sinus by inserting a microhematocrit blood tube into the corner of the eye socket underneath the eyeball and rotate the tube until blood withdraw from retro-orbital vein to fill the tube, directly before both sacrificed which were after 15 and 21 days of treatment for hematological examination according to [11].

The hematological parameters (RBCs and WBCs counts, hemoglobin (Hb)% and packed cell volume (PCV)% were determined at both sacrificed 15 and 21 days post-injection.

### Statistics

Statistical analysis was carried out using one-way analysis of variance (ANOVA). This was done to compare control and treated groups, followed by post-hoc analysis (Dunnett's test) using SPSS (Statistical Package for Social Sciences) version 17 according to [12]. The data were presented in the form of mean ± Standard Deviation. The difference was considered statistically significant if p < 0.05.

### Histopathological examination

All animals were euthanized by injected intraperitoneally (i.p.) with one dose of anesthetized drug combined from (10 mg/kg) xylazine and (100 mg/kg) ketamine, at dose 0.3 ml/rat, after 2 min post-injection, opened the thoracic cavity and insert the 20 gauge needle into the trachea and secure it with a piece of suture then intratracheal inflation of lung with neutral buffered formalin 10% then remove all lungs from the thoracic cavity and full submersion for lung in fixative (neutral buffered formalin 10%), for 72 hr. All fixative lungs of dead and sacrificed animals were embedded in paraffin wax. Sections about 5 μm thickness were prepared and stained with Harries hematoxylin and eosin, and Alcian blue for histopathological examination.

## Results

### Hematological results

The results in Table 1 indicate no statistically significant difference for all parameters in the rats associated with administration of lambda cyhalothrin alone (gp. 2) or lambda cyhalothrin combined with Panax ginseng (gp. 3) for 15 dp.i. in comparison to the control group (gp. 1), while there was a significant difference in the rats which received lambda cyhalothrin combined with garlic (gp. 4) (P value) for red blood cells (P > .049), Hb (P > .037), WBCs (P > .047), and PCV% (P > .025) in comparison with gp.2 (LTC).

**Table 1:** Hematology of the animals treated with normal saline (control) (gp.1), Lambda-cyhalothrin insecticide LTC (gp. 2) and LTC combined with ginseng (gp.3) or garlic (gp.4) in both sacrificed at 15 and 21 d p.i. as short & long term

**Blood parameters No. of animals (10 rats) in each groups No. of animals (10 rats) in each groups**								
**Gps**	**Sacrificed 15 day p.i.**				**Sacrificed 21 day p.i.**			
	**RBC's**	**WBCs**	**Hb.**	**PCV**	**RBC's**	**WBCs**	**Hb.**	**PCV**
	**× 10**^**6**^**/mm3**	**× (10**^**3**^**/mm3)**	**(gm/dl)**	**(%)**	**× 10**^**6**^**/mm3**	**× (10**^**3**^**/mm3)**	**(gm/dl)**	**(%)**
**Control**	7.5 ± 0.36	9.6 ± 1.4	13.58 ± 0.62	34.2 ± 1.9	6.2 ± 0.22	9.9 ± 5.6	12.03 ± 0.76	34.1 ± 3.1
**LTC**	5.3 ± 1.0	8.1 ± 3.4	10.1 ± 1.4	26.4 ± 3.9	3.6 ± 0.79^-a^	16.8 ± 14.7^+a^	8.4 ± 1.27^-a^	21.5 ± 4.39^-a^
**LTC + Ginseng**	5.7 ± 0.53	11.5 ± 1.8	10.7 ± 0.98	28.8 ± 2.4	3.9 ± 0.0^-a^	19.2 ± 0.17^+a^	8.4 ± 0.36^-a^	23.03 ± 0.057^-a^
**LTC + Garlic**	7.0 ± 0.07^+b^	18.1 ± 0.86^+b^	13.0 ± 0.0^+b^	35.7 ± 0.69^+b^	5.2 ± 0.18^+b^	13.4 ± 0.57^-a^	12.2 ± 0.2^+b^	29.7 ± 0.6^+b^

^a^significantly different in comparison with group 1 when p < 0.05.

^b^significantly different in comparison with group 2 when p < 0.05.

However, the animals received LTC and LTC plus Ginseng (gps 2 and 3) treated for 21 days showed a significant difference in red blood cells (P > .001), Hb (P > .007), and PCV% (P > .004), (P > .008), while no statistically significant difference was seen for white blood cell counts in comparison with the control group.

Moreover, the parameters for the rats received lambda-cyhalothrin combined with garlic (gp. 4) were significantly different for red blood cells (P > .015), Hb (P > .019), and PCV% (P > .039) in comparison with gp.2 (LTC), while significantly different for red blood cells (P > .036), Hb (P > .018), and PCV% (P > .094) for comparison with gp.3 (LTC plus Ginseng), but not statistically significantly different for white blood cell counts for most groups.

### Pathological results

Clinical signs included severe coughing, nasal discharge, anorexia, weakness, depression and emaciation in the rats in gp.2 (LTC), which became mild in gp.4 (LTC plus garlic plant). For mortalities, two animals died from gp.2 (LTC), one at the 12th d p.i. and the other at the 17th d p.i. but no mortalities were observed in the remaining groups. Macroscopical findings were congestion and consolidation in the lungs, particularly in gp.2 (LTC).

**Figure 1:**
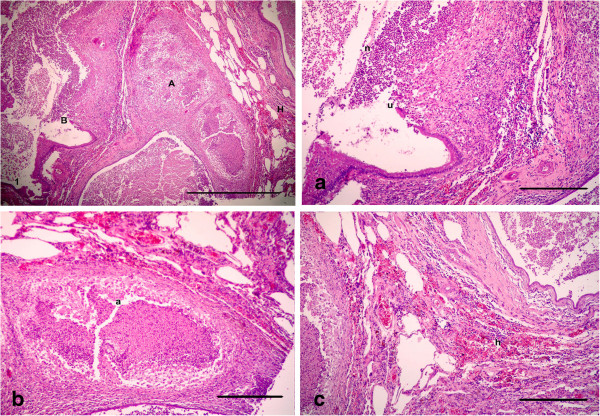
**Photomicrographs of hematotoxylin and eosin-stained sections of the lung in (LTC) group, 15 d p.i. (HE stain).** (1) Bronchiolitis (B), abscessation (A) and hemorrhage (H) (Bar = 200 μm); **(1a, 1b, 1c)** High power of (Figure 1) to show **(1a)** bronchiolitis manifested by hyperplasia with destruction and ulceration (u) in the epithelial lining of the bronchioles replaced by inflammatory cell influx, mainly neutrophils (n), resulting in stenosis and collapse in bronchioles with destruction in the muscles, which were inflamed by aggregation of neutrophils; **(1b)** aggreagation of neutrophils replaced large areas of necrosed pulmonary tissues, forming focal areas of abscessation **(a)** surrounded by fibrous tissues proliferation and congested blood vessels; **(1c)** congestion and hemorrhage (h) among the pulmonary tissues with compensatory emphysema (All Bars = 100 μm).

**Figure 2:**
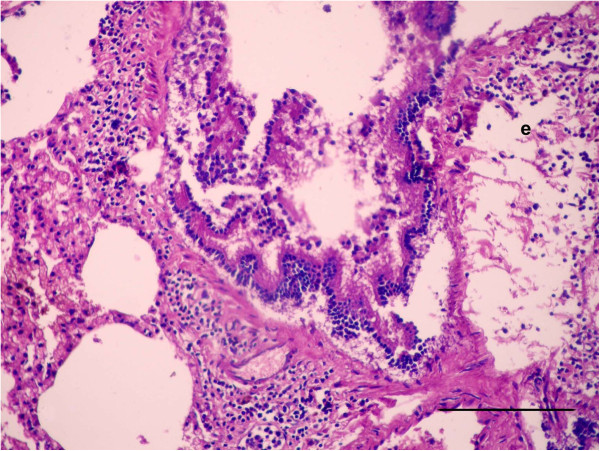
**Photomicrographs of hematotoxylin and eosin-stained sections of the lung in (LTC) group, 15 d p.i. (HE stain).** Hyperplasia with desquamation in the epithelial lining of the bronchioles, with edema in the lamina propria causing detachment of the mucosal layer from the basement membrane, besides inflammatory edema (e) around the bronchioles (Bar = 100 μm).

**Figure 3:**
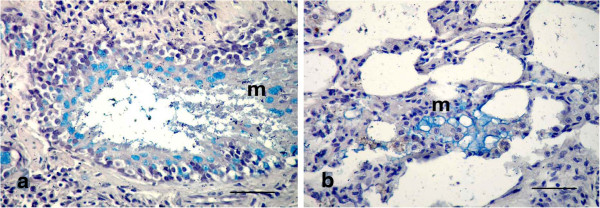
**Photomicrographs of Alcian blue-stained sections of the lung in (LTC) group, 15 d p.i. (Alcian blue stain). (a)** Several goblet cells filled with mucus secretion (m) in the wall of the bronchioles, appearing blue in color; **(b)** Moderate mucus secretion (m) filled the alveoli, appearing blue in color. (All Bars = 50 μm).

For histopathological findings, the animals in group 2 injected with LTC and sacrificed after 15 day of treatment showed bronchiolitis, abscessation and hemorrhage (Figure 1). Same lesions shown in the animals died at the 12th d p.i. Bronchiolitis was manifested by hyperplasia with destruction and ulceration in the epithelial lining of the bronchioles, which were stenosed by inflammatory cell influx of mainly neutrophils, resulting in inflammation with dilation and collapse in the bronchioles, along with destruction and inflammation in muscles by aggregation of neutrophils (Figure 1a). Inflammatory cells, mainly neutrophils, migrated from the inflamed muscle into the peribronchiolar and perialveolar tissues causing inflammation in pulmonary tissues. Aggregation of neutrophils replaced large areas of necrosed pulmonary tissues formed in focal areas of abscessation surrounded by fibrous tissue proliferation and congested blood vessels (Figure 1b). The presence of several areas of abscessation and dilation of bronchioles caused pressure on blood vessels leading to congestion with destruction in some blood vessels, which led to hemorrhage among the pulmonary tissues with compensatory emphysema (Figure 1c). Hyperplasia and desquamation in the epithelial lining of the bronchioles with an increase in mucus secretion inside the lumen, in addition to inflammatory edema in the lamina propria, caused detachment in the mucosal layer from the basement membrane, with mononuclear cells in the wall and around the bronchioles resulting in peribronchiolitis and perialveolitis with compensatory emphysema (Figure 2). Several goblet cells filled with mucus secretion were found in the bronchiole walls (Figure 3a), in addition to moderate mucus secretion filling the alveoli, which appeared blue in color by Alcian blue stain (Figure 3b).

**Figure 4:**
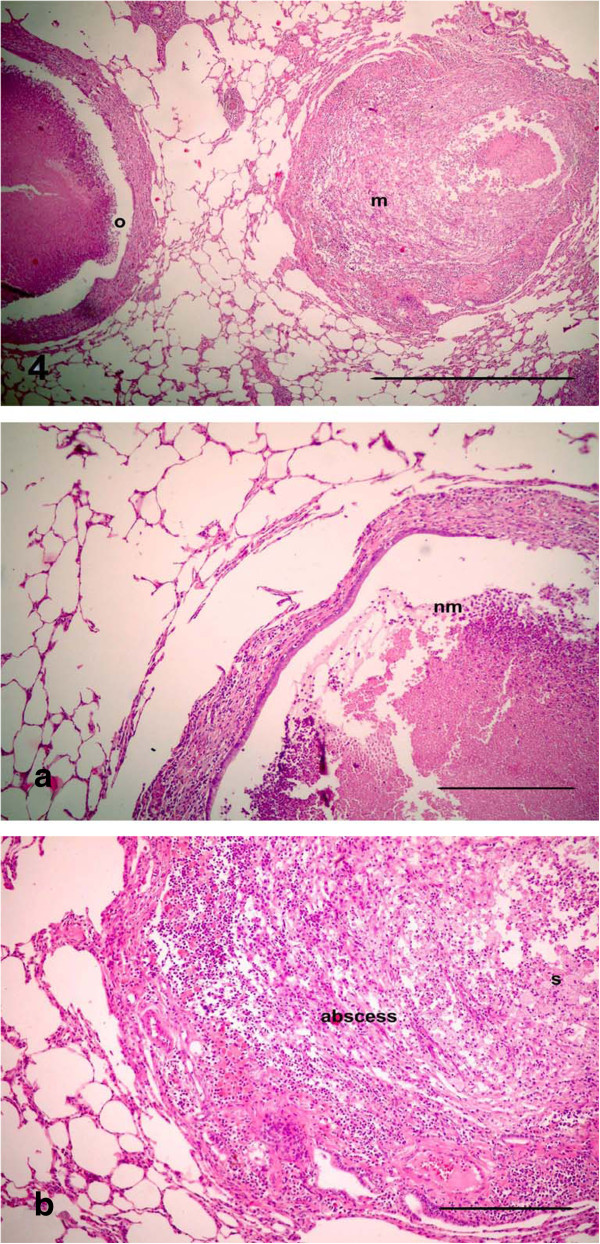
**Photomicrographs of hematotoxylin and eosin-stained sections of the lung in (LTC) group, 21 d p.i. (HE stain).** (4) Obstruction of bronchioles (o) and macroabscessation (m) (Bar = 200 μm); **(4a, 4b)** High power of (Figure 4) to show **(4a)** Obstruction in the bronchioles identified by homogenous material surrounded by live neutrophils mixed with mucus inside the lumen (nm), resulting in blockage in the lumen, with ulceration in the epithelial lining and destruction in the wall; **(4b)** macroabscessation was manifested by a very large circumscribed collection of leukocytes, mainly neutrophils (abscess), with an increase in alveolar secretary cells (s) surrounded by fibrous tissue proliferation replacing the necrosed parenchymatous tissues, with perialveolitis (All Bars = 100 μm).

**Figure 5:**
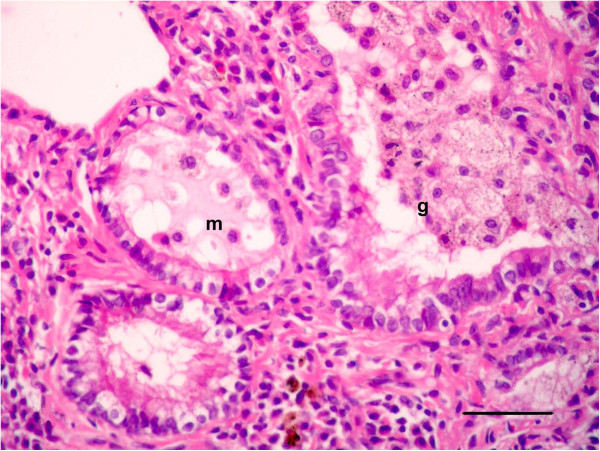
**Photomicrographs of hematotoxylin and eosin-stained sections of the lung in (LTC) group, 21 d p.i. (HE stain).** Obstruction in small and large bronchioles, clarity through the mucus (m) mixed with a few neutrophil cells enclosing the small bronchioles, identified by hyperplasia in the goblet cells, metaplasia (g) accumulating inside the lumen (Bar = 50 μm).

**Figure 6:**
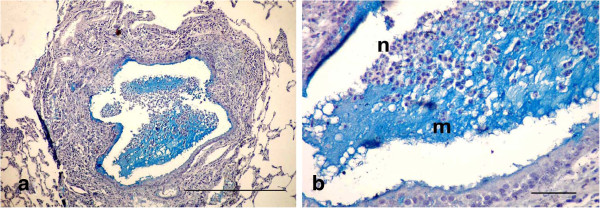
**Photomicrographs of Alcian blue-stained sections of the lung in (LTC) group, 21 d p.i. (Alcian blue stain). (a)** Obstruction in the bronchioles manifested by live neutrophils embedded in the mucus secretion, blue coloration occluding the lumen (Bar = 200 μm); **(b)** High power of (Figure 6a) to show neutrophils (n) embedded in the mucus secretion (m) (Bar = 50 μm).

The dead animals at the 17th d p.i. and sacrificed animals after 21 day of treatment that were injected with LTC showed obstruction of bronchioles and macroabscessation (Figure 4). Obstruction in the bronchioles was identified by homogenous material surrounded by live neutrophils mixed with serous fluid accumulating in the lumen and blocking the lumen, with ulceration in the epithelial lining and destruction in the wall (Figure 4a). Macroabscessation was manifested with a very large circumscribed collection of inflammatory cells, mainly neutrophils, with an increase in alveolar secretary cells surrounded by fibrous tissue proliferation replacing the necrosed parenchymatous tissues, including the perialveolar blood vessels (perialveolitis) (Figure 4b). Obstruction in small and large bronchioles from mucus mixed with neutrophils cells enclosed the small bronchioles (mucinous degeneration), others were identified by hyperplasia of goblet cells accumulating inside the lumen (Figure 5). Interstitial alveolitis with compensatory emphysematous areas were detected. Focal areas of lymphoid cells accumulated nearby blood vessels. Obstruction in the bronchioles was manifested by live neutrophils embedded in the mucus secretion, which appeared blue by Alcian blue stain, and occluded the bronchiole lumen (Figures 6a, b).

**Figure 7:**
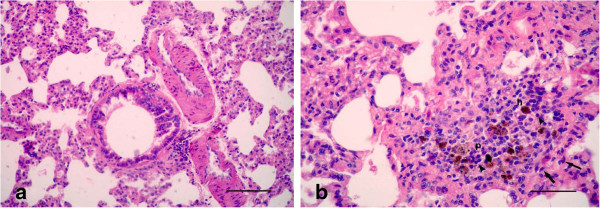
**Photomicrographs of hematotoxylin and eosin-stained sections of the lung in (LTC plus Gensing) group, 15 d p.i. (HE stain). (a)** Moderate hyperplasia in the small bronchioles with a widening in the bronchiolar lumen; **(b)** Focal areas of interstitial pneumonitis (p) was manifested by an aggregation of mononuclear cells (arrowheads) replacing the interstitial tissues with hemosiderosis (h) and fibroblast cells (arrows) (All Bars = 50 μm).

However, most of the animals, which were received panax ginseng plus LTC and were sacrificed after 15 day of treatment showed moderate hyperplasia in the small bronchioles with a widening in the bronchiolar lumen, compared with gp. 2. (LTC) (Figure 7a). Focal areas of interstitial pneumonitis showed an aggregation of mononuclear cells replacing the interstitial tissues with hemosiderosis and fibroblast cells (Figure 7b). Hemosiderosis increased particularly in the areas neighboring the interalveolar blood vessels.

**Figure 8:**
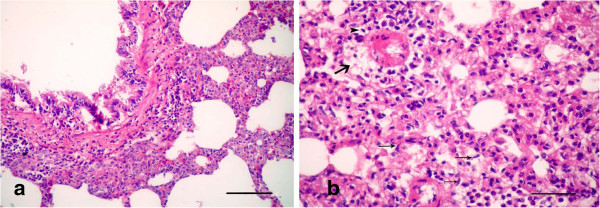
**Photomicrographs of hematotoxylin and eosin-stained sections of the lung in (LTC plus Gensing) group, 21 d p.i. (HE stain). (a)** Mild hyperplasia in the small bronchioles with a normal lumen; **(b)** Alveolitis manifested by edema (arrow) with aggregation of mononuclear cells (arrowhead) around the alveolar blood vessels, with hyperplasia in the alveolar septa, besides pulmonary fibrosis (thin arrows) (All Bars = 50 μm).

**Figure 9:**
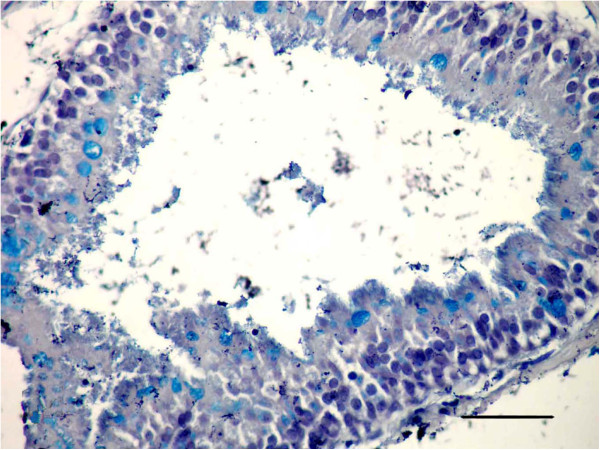
**Photomicrographs of Alcian blue-stained sections of the lung in (LTC plus Gensing) group, 21 d p.i. (Alcian blue stain).** Few goblet cells scavenging the mucus inside the lumen (Bar = 50 μm).

Moreover, mild hyperplasia in the small bronchioles was detected in the animals which were sacrificed after 21 day of treatment (Figure 8a). Alveolitis was manifested by edema with aggregation of mononuclear cells around the alveolar blood vessels, with hyperplasia in the alveolar septa, which characterized the lesions in gp.2 (LTC), in addition to pulmonary fibrosis (Figure 8b). A few goblet cells that scavenge the mucus inside the lumen of the bronchioles appeared with a blue color by Alcian blue stain (Figure 9).

**Figure 10:**
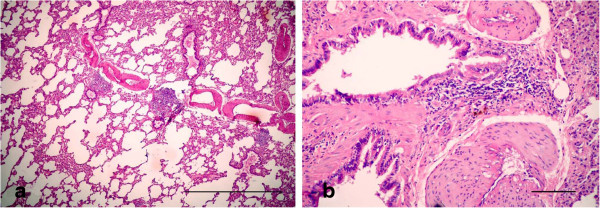
**Photomicrographs of hematotoxylin and eosin-stained sections of the lung in (LTC plus Garlic extract) group, 15 d p.i. (HE stain). (a)** Mild interstitial inflammation and mild hyperplasia shown in the bronchiolar lymphoid follicles (Bar = 200 μm); **(b)** Mild peribronchiolitis manifested by focal areas of round cells accumulating in the wall of bronchioles (Bar = 50 μm).

On the other hand, the animals in group 4 that were given garlic (Allium Sativum) plus LTC sacrificed after 15 day of treatment showed clarified normal lungs with mild interstitial inflammation and mild hyperplasia in the bronchiolar lymphoid follicles (Figure 10a). Mild peribronchiolitis was manifested by normal bronchioles with focal areas of round cells accumulating in the walls of the bronchioles (Figure 10b). Focal areas of chronic inflammatory cells (mainly macrophages, lymphocytes and fibroblasts) replaced the lung tissues near the peribronchioles and perialveolar blood vessels.

**Figure 11:**
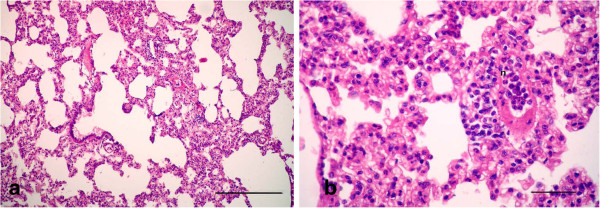
**Photomicrographs of hematotoxylin and eosin-stained sections of the lung in (LTC plus Garlic extract) group, 21 d p.i. (HE stain). (a)** Mild thickening in the alveolar septa (Bar = 100 μm); **(b)** Polymorphonuclear cells, mainly neutrophils, occluding the lumen of blood vessels and decreasing around them, leading to mild alveolitis (Bar = 50 μm).

But the animals that were sacrificed after 21 day of treatment had lung tissues that appeared apparently normal in histology, with mild thickening in the alveolar septa (Figure 11a). Polymorphonuclear cells, mainly neutrophils, occluded the lumen of blood vessels and decreased around them leading to mild alveolitis (Figure 11b).

## Discussion

The present study demonstrated the activation of neutrophils in non-eosinophilic asthma induced by LTC insecticide, and that the use of some plant extracts led to a decrease or disappearance of neutrophils. Our studies revealed no statistically significant difference for all parameters in animals received LTC and LTC plus Ginseng, at 15d p.i. in comparison with the control group, while there was a significant difference in RBCs (P > .001), Hb (P > .007), and PCV% (P > .004), (P > .008), but no statistically significant difference in white blood cell counts in rats treated with LTC and LTC plus Ginseng, at 21d p.i. in comparison with the control group. Meanwhile, all parameters were significantly different for gp 4 (LTC plus garlic), at 15 d p.i., (P value) for RBCs (P > .049), Hb (P > .037), WBCs (P > .047), and PCV% (P > .025) in comparison with gp.2, and returned to normal at 21d p.i. in comparison with the control group.

These results showed that LTC has a toxic effect on the blood while ginseng and garlic were evidently protecting against the asthmatic effect of LTC. Oral exposure to pyrethrins or pyrethroids induces immunosuppressive effects, such as decreased humeral immune response, reduced cell-mediated immune response and leukopenia [13].

The main active components of panax ginseng are ginsenosides, which have a variety of beneficial effects, including anti-inflammatory, antioxidant, and anticancer effects and improved immune function [14]. Panax ginseng (G115) enhances chemotaxis, phagocytosis, increases total lymphocyte count, and increases numbers of T helper cells [15].

Moreover, panax ginseng enhances phagocytosis, natural killer cell activity, and the production of interferon, which causes vasodilation and increases resistance to exogenous stress factors [16,17]. Garlic is useful in preventing the suppression of immune responses [18]. From our results, we suggest that the incomplete improvement effects of ginseng may be due to the high dose of insecticide LTC or from the time of treatment (15 and 21 d p.i.), which was not enough to allow a good recovery.

Our histological findings showed abscess formation characterized by aggregation of neutrophils, which replaced most of the parenchymatous tissues including the bronchiolar lumen, in the group treated with Lambda-cyhalothrin (LTC) (the presence of neutrophils refers to the cases of non-eosinophilic asthma). In addition, secretory cell metaplasia and hyperplasia within the airways induced obstruction of the airways, destruction in some of the bronchioles in addition to congestion and hemorrhage. Lesions were detected in rats that were sacrificed after 15 day of treatment, which appeared more severe in rats sacrificed after 21 day of treatment. Neutrophil elastase also leads to secretory cell metaplasia and hyperplasia within airways, as well as the accumulation of secretory granules [19]. The presence of neutrophils in the sputum is directly correlated with impaired pulmonary function, and suggests a close link between neutrophilic inflammation and airway mucus obstruction [20]. There is a strong association that has been established between neutrophilic inflammation of the airways and severe asthma [21-23]. Neutrophils contain supplies of highly toxic substances, including peroxidases, hydrolytic enzymes, and defensins (antibiotic-like proteins) which they keep in "cytotoxic granules". When they engulf invading cells, they release these granules that poison both the invader and the neutrophils. They can also cause widespread destruction by releasing these granules in a kind of scorched earth policy. Activated neutrophils, monocytes and macrophages all release substances called cytokines. Proinflammatory cytokines help to eradicate infection, remove dead cells and promote tissue repair, such as tumour necrosis factor-α (TNF-α), interleukins 1, 2, 6 and 8 (IL-1, IL-2, IL-6 and IL-8) and interferon-γ., which triggers the release of anti-inflammatory cytokines such as interleukins 4, 10, 11 and 13 (IL-4, IL-10, IL-11 and IL-13), soluble TNF-α and IL-1ra. When the inflammatory trigger subsides, proinflammatory cytokine release stops, allowing the anti-inflammatory cytokines to dominate and restore homeostasis (http://www.mult-sclerosis.org/neutrophil.html). A neutrophilic pattern of inflammation is typically noticed in the bronchoalveolar lavage fluid of patients with chronic inflammatory airway disease, including CF, chronic bronchitis and bronchoectasis [23,24]. Non-eosinophilic asthma represents an alternative asthma phenotype in which patients exhibit asthma symptoms and heightened airway responsiveness in the absence of significant eosinophilia [25].

Our work showed that panax ginseng resulted in moderate hyperplasia in the epithelial lining of the bronchioles with clearance of inflammatory cells and mucus from the lumen, besides moderate interstitial alveolitis with small numbers of neutrophils detected in the rats, particularly those sacrificed after 21 day of treatment when we found a decrease in the sensitivity of the lung against the toxicity of LTC. Other authors have shown that Rhamnogalacturonan II (RG-II) from Panax ginseng regulates the T(H)1/T(H)2 immune response (fate of naive T cells) using an ovalbumin-induced murine model of asthma. They found that in asthma, T helper 2 (T (H)2)-type cytokines such as interleukin (IL)-4, IL-5, and IL-13 are produced by activated CD4(+) T cells. RG-II reduced IL-4 production but increased interferon- gamma production, and inhibited GATA-3 gene expression. RG-II also inhibited asthmatic reactions, including an increase in the number of eosinophils in bronchoalveolar lavage fluid, an increase in inflammatory cell infiltration in lung tissues, airway luminal narrowing, and airway hyper-responsiveness [26]. The inhibitory mechanism of Panax ginseng on asthma parameters in mice is reflected in: PG restoring the expression of estramustine binding protein (EMBP), Mucin-5 subtype AC (Muc5ac), CD40, and CD40L, as well as the mRNA and protein levels of interleukin (IL)-1β, IL-4, IL-5, and tumor necrosis factor (TNF)-α. In addition, PG inhibited the number of goblet cells and in addition small G proteins, cytokines and mitogen activated protein (MAP) kinases in bronchoalveolar lavage cells and lung tissues increased in ovalbumin-induced allergic asthma in mice [27]. Chronic bronchitis treated with antibiotics plus ginseng showed a faster bacterial clearance than those which were treated with antibiotics alone [28].

Our results showed that garlic (Allium Sativum) was more protective against lung asthma, which was evident in the normal bronchiole appearance, with mild interstitial inflammation in all sacrificed rats. The beneficial effects of garlic on asthma are partially due to garlic's ability to inhibit the activity of certain enzymes that generate inflammatory substances. Additionally, fresh garlic is a relatively good source of vitamin C and potassium [29]. Vitamin C helps neutralize free radicals, unstable molecules that cause contraction of airway smooth muscles in asthmatic sufferers and can promote histamine breakdown and reduce histamine release in the body that is generated in many allergic reactions. In addition, garlic can boost the ability of the body to create prostacyclins (lipid molecules) that help keep the air passages of the lungs open and thus promote breathing in asthmatic individuals [29]. Three time repeated intraperitoneal injections of aged garlic extract caused a significant decrease in the hallmark criteria of allergic airway inflammation levels, which included eosinophil percentage in lavage, peribronchial lung eosinophils, IgG1 level in lavage and serum, mucous producing goblet cell grade and peribronchial and perivascular inflammation. This is due to the immunoregulatory effects of aged garlic extract, suggesting a key role for the 14-kD glycoprotein of garlic in shifting the cytokine pattern to T helper-1. The garlic extract includes proteins precipitated by ammonium sulfate, such as IFN-, anti-allergen specific IgE and IgG1 [30].

## Conclusions

It could be concluded that lambda cyhalothrin (LTC), a type of pesticide, caused bronchial obstruction (non-eosinophilic asthma) with abscess formation and interstitial alveolitis in the lungs in all albino rats sacrificed after 15 and 21 day of treatment which was not previously described. In addition, we noticed the incomplete protective effect of ginseng plants against the toxic effect of the pesticides (LTC). Meanwhile, garlic plants showed good results against the severe pathological findings of LTC. This showed that the blockage in the bronchioles in the lung due to LTC showed a broad improvement in easing mild interstitial inflammation, which appeared by treatment with garlic plants more than with ginseng plants.

## Abbreviations

LTC: Lambda cyhalothrin; i.p: Intraperitoneally.

## Competing interests

Neither of the authors has any competing interests.

## Authors’ contributions

All authors gave input to the practical parts, the statistical analysis, interpreted the results and prepared the manuscript. MMM supervised the work and gave input to all areas. All authors read and approved the final manuscript.
